# Adipocyte-Endothelium Crosstalk in Obesity

**DOI:** 10.3389/fendo.2021.681290

**Published:** 2021-08-12

**Authors:** Rugivan Sabaratnam, Per Svenningsen

**Affiliations:** ^1^Steno Diabetes Center Odense, Odense University Hospital, Odense, Denmark; ^2^Section of Molecular Diabetes and Metabolism, Department of Clinical Research, University of Southern Denmark, Odense, Denmark; ^3^Department of Molecular Medicine, Cardiovascular and Renal Research, University of Southern Denmark, Odense, Denmark

**Keywords:** adipose tissue, endothelial cells, hypoxia, extracellular vesicles, endocrine, nitric oxide

## Abstract

Obesity is characterized by pathological adipose tissue (AT) expansion. While healthy AT expansion enhances systemic insulin sensitivity, unhealthy AT expansion through increased adipocyte size is associated with insulin resistance, fibrosis, hypoxia, and reduced adipose-derived adiponectin secretion. The mechanisms causing the unhealthy AT expansion are not fully elucidated; yet, dysregulated crosstalk between cells within the AT is an important contributor. Evidence from animal and human studies suggests a crucial role of the crosstalk between vascular endothelium (the innermost cell type in blood vessels) and adipocytes for metabolic homeostasis. Arterial endothelial cells are directly involved in maintaining normal organ functions through local blood flow regulation. The endothelial-dependent regulation of blood flow in AT is hampered in obesity, which negatively affects the adipocyte. Moreover, endothelial cells secrete extracellular vesicles (EVs) that target adipocytes *in vivo*. The endothelial EVs secretion is hampered in obesity and may be affected by the adipocyte-derived adipokine adiponectin. Adiponectin targets the vascular endothelium, eliciting organ-protective functions through binding to T-cadherin. The reduced obesity-induced adiponectin binding of T-cadherin reduces endothelial EV secretion. This affects endothelial health and cell-cell communication between AT cells and distant organs, influencing systemic energy homeostasis. This review focuses on the current understanding of endothelial and adipocyte crosstalk. We will discuss how obesity changes the AT environment and how these changes contribute to obesity-associated metabolic disease in humans. Particularly, we will describe and discuss the EV-dependent communication and regulation between adipocytes, adiponectin, and the endothelial cells regulating systemic energy homeostasis in health and metabolic disease in humans.

## Introduction

Obesity, defined as excessive fat accumulation, is a worldwide epidemic accompanied by an increased risk of developing cardiovascular diseases (CVDs), certain types of cancers, Alzheimer’s disease, non-alcoholic fatty liver disease, and type 2 diabetes mellitus (T2D) (1). The link between the increased disease risks and excessive fat accumulation is not completely understood but appears to rely on impaired white adipose tissue (WAT) function. WAT functions as an energy buffer that stores and releases energy (2); however, WAT is also an essential endocrine organ and plays a key role in regulating systemic glucose and energy metabolism by secreting an array of adipokines, including leptin and adiponectin (3). WAT is recognized as a highly dynamic and heterogeneous organ, and the adaptation to expanding WAT requires coordinated actions of multiple cell types to ensure a healthy adipocyte environment.

The WAT adapts to the excessive energy intake through two mechanisms 1) an increase in adipocyte number (hyperplasia) and/or 2) size (hypertrophy) (4). The hyperplastic WAT expansion is characterized by the formation of new adipocytes from adipose progenitor cells, which is associated with enhanced systemic insulin sensitivity. Hypertrophic WAT expansion is, on the other hand, characterized by insulin resistance, dysfunctional prolipolytic action, increased inflammation, fibrosis, and altered adipokine secretion profile, including decreased adiponectin levels (4, 5). Unhealthy WAT expansion is the *sine qua non* of metabolic unhealthy obesity, causing ectopic lipid accumulation in peripheral tissues such as the liver and skeletal muscle (4). The molecular mechanisms underlying the transition from a healthy WAT to an unhealthy pathological expansion are yet to be elucidated. Here, we will review and summarize our current understanding of the crosstalk between the adipocytes and the arterial endothelial cells within the WAT and how this communication potentially regulates systemic energy homeostasis in metabolic disorders.

## The Adipose Tissue Microenvironment

Adipose tissue is a heterogenous cell population and contains, besides adipocytes, fibroblasts, stem cells, immune cells, and endothelial cells, and their intercellular crosstalk is crucial for the microenvironment (6). There is substantial evidence, mainly from animal studies, that hypertrophic obesity is associated with low oxygen tension in AT and increased expression of hypoxia-response genes (7), including the master regulator of hypoxia, hypoxia-inducible factor 1 (HIF1) (8). In primary adipocytes and macrophages from lean mice, hypoxia increases expression of inflammatory markers such as TNF-α, IL-1, IL-6, and TGF-β, chemokine (MIF), extracellular enzyme (MMP9), and macrophage markers (CD11 and F4/80) (7). The effect of hypoxia on the expression of adipokines in human adipocytes revealed increased gene expression levels of FIAF/angiopoietin-like protein 4, IL-6, leptin, MIF, PAI-1 and vascular endothelial growth factor (VEGF) (9). In 3T3-L1 cells, the promoter activity of NFkappaB and TNF-α was activated by hypoxia, causing reduced adiponectin promoter activity (7). Adipocyte-specific knockout of Hif1β – the obligate partner of hypoxia-inducible factors (Hif1a, Hif2a, and Hif3a) - reduced weight gain relative to wild-type controls and showed decreased high-fat diet (HFD)-induced obesity and glucose intolerance (10), indicating that the adipocyte-response to hypoxia is negative.

Oxygen tension in human WAT is challenging to measure, and the measured levels between lean and obese are dependent on the techniques used (11). Goossens et al. found that oxygen tension in WAT was higher in obese, and the obese subjects had lower WAT oxygen consumption (12). On the other hand, Pasarica et al. found that obesity was associated with lower WAT partial O_2_ pressure, and obese subjects had a lower capillary density and decreased gene expression of WAT VEGF (13). Despite these differences in these two studies, the obese subjects had insulin resistance, high expression of inflammatory cell markers, and lower WAT capillarization (12, 13). Cifarelli et al. reported that the human AT expansion in obesity is associated with reduced AT pO2, which contributes to increased AT HIF-1α expression (14). These alterations decrease the branched-chain amino acid (BCCA) catabolism and increase the AT inflammation and fibrosis. Ultimately, this leads to an increase in circulating BCAAs and PAI-1 causing systemic insulin resistance (14). Very recently, Todorčević et al. demonstrated that markers of subcutaneous AT hypoxia are elevated in severely obese patients with obesity hypoventilation syndrome but not in moderately obese individuals, suggesting that in moderate obesity, AT dysfunction may not be driven by hypoxia (15).

Capillarization is endothelial-dependent, and blockage of vascularization in WAT causes unhealthy tissue expansion, enhanced inflammation and fibrosis, leading to systemic insulin-resistance (16–21). On the other hand, stimulation of angiogenesis results in healthy WAT expansion even during HFD-feeding and is associated with maintained insulin-sensitivity (16–21). Collectively, it appears that the interplay between AT cells is crucial for metabolic homeostasis and hampered endothelial-dependent regulation of WAT blood flow affects AT plasticity.

Using single-cell RNA-sequencing (scRNA-seq), it has become possible to perform large-scale transcript profiling of heterogeneous cell populations obtained from WAT from mice (22, 23) and humans (24). scRNA-seq of the stromal vascular fractions from visceral and subcutaneous WAT samples from obese patients undergoing bariatric surgery classified the cells into three subpopulations: 1) progenitors or stem cells (55%), 2) immune cells (37%), and 3) endothelial cells (8%) (24). Interestingly, endothelial cells could furthermore be divided into three types of endothelial cells (EC1-3). EC1 cells express genes (*FABP4*, *LGALS1*, *RBP7*, *GPX3*, and *CD36*) involved in lipid handling machinery, while EC2 cells had pronounced expression of canonical endothelial markers (*ACKR1*, *SELE*, *TM4SF1*, *VCAM1*, *TMEM173*, *PLVAP*, *ICAM1*, *PECAM1*, *VWF*, *ADAMTS9*, and *TFPI*). EC3 cells were highly enriched in LYVE1 expression, which is a marker of lymphatic endothelial cells (24). The lymphatic endothelial cells were predominantly present in visceral WAT samples (24). Although it is beyond the present review’s scope, it is worth mentioning that lymphatic vessels (25) and immune cells (26) have been shown to contribute to unhealthy obesity. For example, lymphatic vasculature dysfunction was associated with an adult-onset obesity phenotype (27), and ablation of macrophages, through transgenic expression of diphtheria-toxin receptor under control of CD11c promoter, in mice fed an HFD normalized insulin sensitivity and reduction in local and systemic inflammation markers (28). Nonetheless, the vascular endothelial cells, representing only a minor fraction of the total cell population, have prominent physiological and biological roles in health and metabolic disease. Importantly, endothelial dysfunction is an early vascular abnormality in metabolic disorders, and emerging evidence supports a critical role of endothelial cells in the development of metabolic disorders.

## Vascular Endothelial Cells Are Crucial for Whole-Body Metabolism

The vascular endothelial cells are the inner most cell type of arteries, veins and capillaries. The vascular endothelial cell function sustains organ homeostasis by regulating vascular tone, recruitment of blood cells, exchanging tissue factors, forming new blood vessels, and providing organ-specific barrier function (29). Unlike other healthy cell types, endothelial cells generate most of their ATP from glycolysis (30). Endothelial cells have an insulin-independent glucose uptake through glucose transporter 1 (GLUT1) (31), and hyperglycemia, an associated consequence of obesity, is likely to increase the endothelial cells glucose concentration, which in itself is enough to cause oxidative stress and endothelial dysfunction (32). Although the endothelial glucose uptake is insulin-independent, impaired insulin signaling in endothelial cells can affect systemic insulin sensitivity. HFD fed mice have a reduction in insulin receptor substrate (Irs) 1 and 2 in the endothelial cells, and endothelial-specific knockout of Irs2 impaired insulin-induced glucose uptake in skeletal muscle (33). A vital function of the endothelium is to induce relaxation of the underlying vascular smooth muscle cells through the release of nitric oxide (NO) and thereby increase blood flow. The endothelium NO is generated primarily by the endothelial NO synthase (eNOS), and in endothelial-specific Irs2 knockout mice, the insulin-induced eNOS activation by phosphorylation was blocked, likely through reduced phospho-Akt (33). eNOS-phosphorylation was restored by the stable prostaglandin I2 analog beraprost sodium, which reestablished glucose uptake by the skeletal muscle in the endothelial Irs2 knockouts on a normal and HFD (33). Moreover, the antidiabetic drug metformin, which enhances whole-body insulin sensitivity, improves endothelial-dependent relaxation (34), indicating a critical role for endothelial blood flow regulation for the AT.

## Endothelial-Dependent Blood Flow

The endothelial-dependent vasodilatation is compromised in metabolic diseases, including obesity, and aging and is linked to reduced release of NO (35), increased oxidative stress (36), decreased endothelium-dependent hyperpolarization (37), and upregulation of released endothelium-derived contracting factors (38), which in combination results in reduced relaxation to vasodilatory substances such as acetylcholine in *ex vivo* arterial preparations. In humans and mice, increased body weight is associated with decreased endothelial-dependent vasodilatory in response to vasodilatory substances (39–43). Ingestion of food increases adipose tissue blood flow (ATBF) (44), and an oral glucose load increased ATBF in lean but not in obese subjects (12). The mediators responsible for this fast adjustment of ATBF are unknown, but intervention studies have provided evidence for the factors involved. Although increased glucose levels enhance plasma insulin levels, insulin micro infusion did not directly affect ATBF in humans (45). However, the ATBF increase correlated with changes in plasma norepinephrine (45). Plasma norepinephrine is likely to derive from spill over of sympathetic activity in muscle and AT (46). Norepinephrine is an endothelium-dependent arterial vasodilator (47) and high ATBF responding subjects had greater changes in plasma norepinephrine (44). Moreover, pharmacological intervention studies in humans have demonstrated that ATBF relies on the endothelial NO system (11, 48, 49), indicating that sympathetic activity is involved in ATBF regulation through an endothelial-dependent mechanism in arteries. HFD treatment of mice causes a reduced eNOS expression in WAT and transgene eNOS overexpression in whole body endothelial cells protected against high-fat diet-induced obesity (50). In agreement, eNOS deficient mice exhibit systemic insulin resistance (51, 52). Although the genetic interventions are not specific to WAT endothelium but affects all endothelial cells, the data suggest an essential role of eNOS regulation and endothelial health.

We have recently shown that mice fed an HFD developed endothelial dysfunction, which was abrogated in mice with global knockout of the T-type Ca^2+^ channel Cav3.1 (53). Consistent with this, in hypertensive patients treated with T-type/L-type channel blocker Efonidipine improved endothelial function (54), and pharmacological and genetic inhibition of Cav3.1 likewise protects against HFD-induced obesity in mice (53, 55). Cav3.1 is, among others, expressed in AT and endothelial cells (53), but the pharmacological and genetic inhibition studies of Cav3.1 do not pinpoint which cell types are involved in the phenotype. In endothelial cells, Cav3.1 interacts with eNOS (56). Nonetheless, global Cav3.1 knockout mice fed a regular diet display eNOS activity both *in vivo* and *in vitro* (56), and how Cav3.1 deficiency affects eNOS activity in HFD mice is still not known.

eNOS activity is also affected by caveolin-1 (Cav-1) – an integral membrane protein critically involved in the invagination of caveolae from the plasma membrane. Global Cav-1 knockout mice have endothelial dysfunction (57), and transgenic Cav-1 re-expression in the endothelium of Cav-1 knockout mice rescues the endothelial function (58). Cav-1 inhibits eNOS (59) and HFD-induced obesity increases vascular Cav-1 expression and accompanies impaired NO-mediated vasodilatation (60). In summary, the data suggest that adequate eNOS regulation in AT endothelial cells is important for ATBF and whole-body energy homeostasis, and that Cav-1 appears to play a significant role.

Cav-1 is not only expressed in vascular endothelial cells but also highly expressed in adipocytes. Recently knockout of the Cav1 gene in mice uncovered a significant extracellular vesicle (EV)-mediated signaling between endothelial cells and adipocytes (61).

## Extracellular Vesicles Are Involved in Endothelial-Adipocytes Crosstalk

The term EV encompasses several distinct vesicle types but can broadly be divided into microvesicles and exosomes (62). Microvesicles originate from the plasma membrane through outward budding, while exosomes are created through invagination of the plasma membrane that ultimately causes the formation of multivesicular bodies (MVB), which through fusion with the plasma membrane release exosomes to the extracellular medium. The EVs all contain constituents of a cell, including nucleic acid, lipids, and nuclear, cytosolic, and membrane proteins (63, 64). Although the function(s) of EVs are still unknown, the fact that all cells, pro- and eukaryotes (65), release EVs points to their contribution to normal physiology, and EV appears to be involved in cell-cell communication and cellular waste management.

### Extracellular Vesicles and Cell-Cell Communication

EVs have been suggested as entities for horizontal transfer of genetic material and proteins between cells. RNA is the dominant form of nucleic acid in EVs, and EVs appear to be enriched for several specific RNA species, including a number of microRNAs (miRNAs). In agreement, it was detected early that the correlation between cellular and EV RNA concentrations was poor (66), hinting at an active transport of RNA molecule into vesicles. Indeed, a short RNA motif has been identified that guides RNA into EVs (67). Nonetheless, using the golden standard for EV isolation – differential ultracentrifugation – has revealed that the average number of miRNAs per EV is low – approximate 1 miRNA per 100 EV (68). This low RNA/EV ratio suggests that EVs and their cargo may be heterogeneous and that some EVs carry a lot of RNA while others are non-RNA-carriers. Consistent with this, new EV separation and isolation techniques such as asymmetric-flow field-flow fractionation indicate that there exists a much wider variety of EVs than has previously been recognized (69). It should be noted that the analysis of EVs is complicated, and current EVs isolation techniques carry a significant risk of analysis of contaminations such as co-precipitated RNAs and proteins (62). This is even true for EVs isolated from serum-free medium where supplements may carry a significant amount of miRNA (70). Interestingly, though, in humans and mice, adipocyte-derived EV contain a significant fraction of circulating miRNAs (71). This was determined using mice with adipocyte-specific knockout of Dicer, a critical enzyme required for the conversion of pre-miRNA molecules into a mature miRNA (71). Disruption of the adipocyte processing of miRNAs caused significantly reduced plasma EVs level of miRNAs (71). The circulating adipocyte EV reduced hepatic FGF21 expression causing a decreased plasma FGF21 level and improved glucose tolerance, indicating that the EV miRNAs were functional (71). Collectively, it appears that adipocyte-derived EVs mediated cell-to-cell communication that affects distant organ function and surrounding cells.

Hypoxia is a potent stimulator of EV secretion. EVs released from adipocytes cultured at 1% O_2_, compared to normal air with 5% CO_2_, and EVs from obese subjects impaired insulin-stimulated glucose uptake in adipocytes (72). Moreover, in the initial stages of HFD-induced AT expansion, the increased AT oxygen consumption limits O_2_ availability imposing a state of relative AT hypoxia that stimulated VEGF expression, and increased angiogenesis and HIF1α expression (73). In 3T3-L1 adipocytes, proteomic analysis of the EVs from the normoxic (20% O_2_) and low oxygen (1% O_2_) cultured 3T3-L1 cells showed that 75 and 67 proteins were up- and down-regulated, respectively, by the low oxygen conditions and that the EVs were enriched in proteins involved in *de novo* lipogenesis (74). Importantly, the low oxygen-derived EVs promoted the accumulation of lipids in recipient cells (74). EVs from liver cells are also important for lipid accumulation. Mice fed an HFD display rapid lipid accumulation in the liver (within hours), and the liver has been shown to respond to this by increased EV secretion (likely exosomes), which target adipocytes (75). Inhibition of EV secretion from liver cells by knockdown of Geranylgeranyl diphosphate synthase (Ggpps) improved glucose tolerance in HFD-fed mice but did not improve insulin resistance. The liver-derived EVs enhance adipocyte lipid deposition by increasing lipogenesis and inhibiting lipid oxidation through Pgc1α. Thus, liver cells may be an early metabolic sensor of lipid overload and respond by increased EV signaling to adipocytes (75).

In the WAT, the adipocytes are also targeted by EV derived from endothelial cells (61). Endothelial-derived EVs transferred Cav-1 protein to adipocytes, and, importantly, the EV-mediated transfer was regulated by fasting and feeding (61). Fasting increased endothelial Cav1 transfer, and this effect was blunted in HFD treated and *ob*/*ob* mice (61). Although the glucagon receptor expression did not differ between WAT- and lung-derived endothelial cells (61), glucagon only increased endothelial-derived EV secretion from WAT-derived endothelial cells (61). An essential function of the endothelial cells is the transcytosis of plasma components to the underlying parenchyma (29). Crewe et al. found that cultured endothelial cell-derived EVs were enriched in FBS-derived protein components (61). Glucagon and insulin increased BSA (fatty acid-free, low endotoxin) uptake in cultured endothelial cells, and Cav-1 participated in the process but was not essential for the glucagon and insulin-stimulated BSA uptake in endothelial cells (61). The BSA was secreted in EVs and indicates that endothelial cells contribute significantly to transcytosis by uptake of plasma components and secretion in EVs (61). Thus, the intercellular and interorgan EV communication to and from the WAT appears to be important for metabolic regulation.

### Extracellular Vesicles as Cellular Waste Management

Accumulating evidence suggests that EVs are part of the cellular waste management system and shares many features with secretory autophagy (76). For example, blockage of EV secretion causes accumulation of harmful DNAs and activation of cellular damage response (77). The important role of autophagy for adipocytes and endothelial cells has been demonstrated in experimental models. Mice with the adipocyte-specific knockout of autophagy genes Atg3 and Atg16L had normal weight and body composition; however, the gene disruptions caused a massive influx of inflammatory cells in AT even in the regular diet-fed mice (78). This occurred without an increase in cytokines such as TNF-α, IL-6, or MCP1 (78). The knockouts developed insulin resistance and impaired glucose tolerance, and together, suggests an essential role for adipocyte autophagy in the development of insulin resistance independent of obesity (78). Autophagy is also crucial for endothelial cells. Obesity-induced endothelial dysfunction is associated with the upregulation of endothelial autophagy machinery (79) and vascular ceramide content (80). Exosome production and release are modified by ceramide synthesis (81), and, interestingly, adiponectin signaling in endothelial cells increased exosome secretion and reduced cellular ceramide levels (74). Thus, exosome secretion may be a critical mechanism to reduce the intracellular accumulation of toxic material and endothelial dysfunction through adipocyte secretion of adiponectin.

## Adiponectin - T-Cadherin-Axis and Cellular Crosstalk

Adiponectin is an adipokine, which acts in an autocrine/paracrine and endocrine fashion (82) and is highly expressed in human and mouse AT (82, 83). Various adipokines may play a key role in AT biology, on systemic metabolism or tissue crosstalk such as leptin, however, in this review we are only focusing on adiponectin. Typically, plasma concentration of adiponectin is high and in the micromolar range. Low adiponectin levels are reported in humans with metabolic diseases such as obesity and T2D (84) and are inversely correlated with insulin resistance (85) and fat mass in humans (86). Moreover, decreased levels of adiponectin are also reported in coronary artery disease (87) and myocardial infarction (88). Thus, reduced circulating adiponectin levels can reflect metabolic perturbations and can potentially serve as a critical marker of WAT fitness.

Adiponectin belongs to the C1q-like superfamily of protein, and its structure consists of a 22 collagen repeats and a C-terminal C1q-like globular domain (82). Endogenous adiponectin forms homo-oligomeric structures consisting of trimers, dimers of trimers, and 4- and 5-mers of trimers that is referred to as low molecular weight (LMW), medium molecular weight (MMW), and high molecular weight (HMW), respectively, complexes (89, 90) with different biological functions through binding of surface receptors (91–93).

Activation of the adiponectin receptors AdipoRs (94–96) and calreticulin (91) have important metabolic and immunological roles. Skeletal muscle is an important site of insulin-mediated glucose uptake; thus, considerable emphasis was placed on studying the possible metabolic effects of adiponectin on muscle. In cultured muscle cell lines, adiponectin improves insulin sensitivity (97), increases glucose uptake (63, 98) and increases fatty acid oxidation (63, 64). In mouse models of obesity and T2D, physiological doses of adiponectin enhanced insulin sensitivity (99). In muscle, adiponectin acts through AdipoR1 to activate AMPK (100). The anti-inflammatory effects of adiponectin have been demonstrated in different cell studies. Treatment of human macrophages with adiponectin revealed that adiponectin inhibits mature macrophages’ phagocytic activity, and adiponectin also inhibited the lipopolysaccharide (LPS)-induced TNF-α production and TNF-α mRNA expression (101). In line with this, another study showed that treatment of peritoneal macrophages with recombinant adiponectin enhanced transcript levels of a marker of the M2 phenotype such as IL-10 (102), indicating that adiponectin promotes macrophage polarization toward an anti-inflammatory M2 phenotype. In the liver, adiponectin binds to AdipoR1 and AdipoR2 to suppress hepatic glucose production and glycogenolysis (103), leading to reduced plasma glucose levels. Decreased hepatic glucose production can possibly be explained by studies showing that adiponectin suppresses the key regulators involved in gluconeogenesis, including phosphoenolpyruvate carboxykinase and glucose-6-phosphatase (104, 105).

In addition to adiponectin’s beneficial effects on muscle and liver, it is also protective through its effects on the vascular endothelium (106). In the vascular endothelium, adiponectin acts through AdipoR1 and AdipoR2 to increase NO production through AMPK, which activates eNOS, leading to vasodilation (106). In addition to AdipoR1 and AdipoR2, endothelial cells also bind adiponectin through T-cadherin expression.

As described above, adiponectin increases endothelial exosome secretion, and this effect is mediated through the binding of adiponectin to T-cadherin (107). T-cadherin is a member of the cadherin family, but in contrast to the other family members, it lacks a C-terminal intracellular domain and is attached to the extracellular side of the plasma membrane through a glycosylphosphatidylinositol (GPI)-anchor (108). There is strong *in vivo* support for the adiponectin/T-cadherin interaction from human and murine studies. Thus, genome wide-association studies (GWAS) for plasma adiponectin levels suggest the CDH13 (the gene encoding T-cadherin and outside of the *ADIPOQ* locus) is strongly linked to adiponectin levels (109), and CDH13 single nucleotide polymorphisms (SNPs) are linked to increased adiponectin plasma levels in humans (110, 111). In mice, T-cadherin deficiency causes 3-fold increased plasma adiponectin levels (112). T-cadherin is expressed in the heart, skeletal muscle, aorta, and vascular endothelium (112–114). Adiponectin in its hexameric and HMW – but not trimeric and globular-forms bind T-cadherin (92, 115), and the T-cadherin expression tissues are also the site for the accumulation of HMW and hexameric adiponectin in mice (112–114, 116, 117), suggesting critical biological functions of the adiponectin binding to T-cadherin. Adiponectin and T-cadherin knockout mice have lower plasma exosome levels, and viral overexpression of adiponectin caused increased plasma exosome levels (107). The mechanisms and cellular signaling pathways that are involved are still unknown. It has, however, been shown that oligomerization of membrane-anchored stimulates their sorting of cargo into exosome (118, 119); thus, the binding of the cellular attached T-cadherin to adiponectin might cause T-cadherin oligomerization, internalization, and sorting into the multivesicular bodies (MVBs) before being released as exosomes. Thus, the crosstalk between adipocytes and endothelial cells involves EVs; however, this crosstalk’s biological significance is still largely unknown but could be an important mechanism mediating tissue-crosstalk and endothelial health. Further research should be undertaken to investigate if other adipokines also play a key role in crosstalk mediated by EVs.

## Concluding Remarks and Future Perspectives

**Figure 1:**
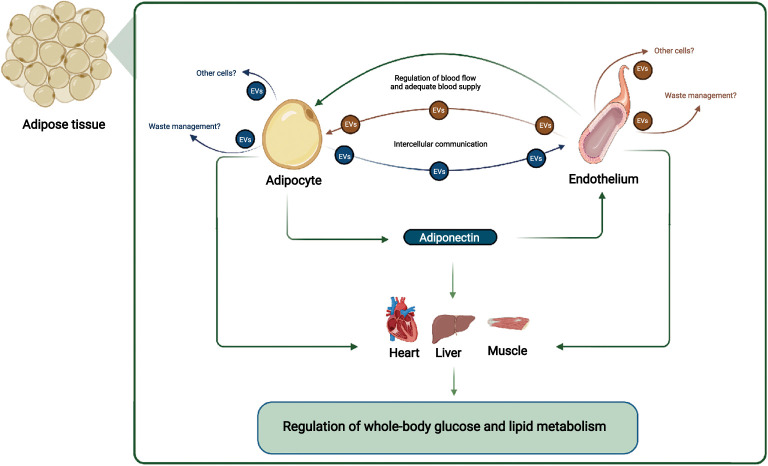
Adipocyte and endothelial crosstalk in adipose tissue contribute to regulation of whole-body glucose and lipid metabolism: In this working hypothesis, endothelial cells regulate blood flow adequately to adipocytes, which secrete adiponectin that communicate locally with endothelial cells and with distant organs such as muscle, liver, and heart. Adiponectin stimulates EV secretion from endothelial cells, ensuring their health e.g. through cellular waste management, and stimulates muscle, liver, and heart metabolism, improving systemic metabolism. EVs from adipocytes and endothelial cells may also target other cell types. Other adipocyte-derived factors, and overall endothelial health also contributes to regulation of whole-body glucose and lipid metabolism. Figure created with BioRender.com.

The WAT microenvironment is critical for a whole-body metabolism; thus, gaining a mechanistic understanding of the crosstalk between the different cell populations in WAT is crucial. The endothelial cells are important for regulating WAT blood flow, and inadequate blood flow may cause hypoxia in the WAT and reduced adiponectin secretion (**Figure 1**). The reduced adiponectin secretion may cause reduced EV secretion from endothelial cells and thereby accelerate the development of endothelial dysfunction and decreasing adipocyte function further (**Figure 1**). The reduced adipocyte function affects multiple organs, e.g., through decreased adiponectin signaling in skeletal muscle, liver and the heart, and targeted treatment that restores the adipocyte/endothelial crosstalk in WAT may thus provide therapeutic opportunities that improve whole-body metabolism.

Since the crosstalk involves physiological adaptations, such as changes in blood flow, and molecular changes, the investigations are heavily dependent on integrated models. In line with this, the exact role of EVs for WAT biology regulation also requires integrated models and needs the development of new experimental tools. The effect of EVs is often inferred from correlation studies, and tools that block exosome signaling in a cell-specific manner are still not available. The use of fluorescent-tagged proteins that are transferred from endothelial cells to adipocytes has convincingly shown that transport does indeed occur between different cell types *in vivo* (61); however, to obtain information on EV function directly, cell-specific manipulation of EVs needs to be established. Possible solutions could be to use single-cell assays such as CD63-pHluorin (120, 121) and *in vivo* models for tracking intercellular EV communication (122). The tagged EVs will allow for cell-specific quantification of EV release rate and enable the identification of genetic and pharmacological interventions that interferes with EVs.

From a translational perspective, cells – including adipocytes and endothelial cells – release EVs to the circulation and provide non-invasive access to organs within the body. We have shown, using paired samples of human kidney and urine samples, that the EVs protein abundance is not a reliable marker of its tissue abundance (123). Nonetheless, the AT-derived EVs may be used to monitor clinical intervention studies and for early detection and differentiating of individual subjects based on whether or not they have healthy or unhealthy obesity. The identification of adipocyte- and endothelial-specific EV markers will provide an approach that enables the non-invasive interrogation of crosstalk between the cell-types in humans, enabling the translation of the findings from animal models to humans, and thereby provide new treatment options for alleviation of the negative health impacts of obesity.

## Author Contributions

RS prepared the figure. RS and PS drafted the manuscript. RS, PS revised the manuscript. Both authors contributed to the article and approved the submitted version.

## Funding

Steno Diabetes Center Odense funded by the Novo Nordic Foundation (RS), and the Novo Nordisk Foundation (grant no NNF20OC0063791 to PS).

## Conflict of Interest

The authors declare that the research was conducted in the absence of any commercial or financial relationships that could be construed as a potential conflict of interest.

## Publisher’s Note

All claims expressed in this article are solely those of the authors and do not necessarily represent those of their affiliated organizations, or those of the publisher, the editors and the reviewers. Any product that may be evaluated in this article, or claim that may be made by its manufacturer, is not guaranteed or endorsed by the publisher.
